# Direct free radical scavenging effects of water-soluble HMG-CoA reductase inhibitors

**DOI:** 10.3164/jcbn.18-48

**Published:** 2018-08-08

**Authors:** Ryohei Umeda, Hiroki Takanari, Kazue Ogata, Shigekiyo Matsumoto, Takaaki Kitano, Katsushige Ono, Osamu Tokumaru

**Affiliations:** 1 Department of Pathophysiology, Oita University Faculty of Medicine, 1-1 Idaigaoka, Hasama-machi, Yufu, Oita 879-5593, Japan; 2 Clinical Research Center for Diabetes, Tokushima University Hospital, 2-50-1 Kuramoto-cho, Tokushima 770-8503, Japan; 3 Department of Anesthesiology, Oita University Faculty of Medicine, 1-1 Idaigaoka, Hasama-machi, Yufu, Oita 879-5593, Japan; 4 Department of Physiology, Faculty of Welfare and Health Sciences, Oita University, 700 Dan-noharu, Oita 870-1192, Japan

**Keywords:** water-soluble statin, electron spin resonance spectrometry, free radical, fluvastatin, pravastatin

## Abstract

3-Hydroxy-3-methylglutaryl coenzyme A reductase inhibitors, statins, are widely used for preventing cardiovascular and cerebrovascular diseases by controlling blood cholesterol level. Additionally, previous studies revealed the scavenging effects of statins on free radicals. We assessed direct scavenging activities of two water-soluble statins, fluvastatin and pravastatin, on multiple free radicals using electron spin resonance spectrometry with spin trapping method. We estimated reaction rate constants (*k*_fv_ for fluvastatin, and *k*_pv_ for pravastatin). Superoxide anion was scavenged by fluvastatin and pravastatin with *k*_fv_ and *k*_pv_ of 4.82 M^−1^s^−1^ and 49.0 M^−1^s^−1^, respectively. Scavenging effects of fluvastatin and pravastatin on hydroxyl radical were comparable; both *k*_fv_ and *k*_pv_ were >10^9^ M^−1^s^−1^. Fluvastatin also eliminated *tert*-butyl peroxyl radical with relative *k*_fv_ of 2.63 to that of CYPMPO, whereas pravastatin did not affect *tert*-butyl peroxyl radical. Nitric oxide was scavenged by fluvastatin and pravastatin with *k*_fv_ and *k*_pv_ of 68.6 M^−1^s^−1^ and 701 M^−1^s^−1^, respectively. Both fluvastatin and pravastatin had scavenging effects on superoxide anion, hydroxyl radical and nitric oxide radical. On the other hand, *tert*-butyl peroxyl radical was scavenged only by fluvastatin, suggesting that fluvastatin might have more potential effect than pravastatin to prevent atherosclerosis and ischemia/reperfusion injury via inhibiting oxidation of lipids.

## Introduction

Excessive accumulation of reactive oxygen species or free radicals increases the risk for various cardiovascular and cerebrovascular diseases.^(1,2)^ One of the major mechanisms of such pathology is atherosclerosis. Recent basic researches demonstrated that oxidized low-density-lipoprotein accelerated the formation of atherosclerosis.^(3)^ It was shown that metabolic abnormalities of lipids such as cholesterol and free fatty acid (FFA) correlated with the prognosis of whole body ischemia/reperfusion (I/R).^(4,5)^ These findings suggested that lipid oxidation is closely involved in various pathological conditions. Research and development of drugs to prevent the production of free radicals or oxidation of lipids have been conducted in recent years. Edaravone is the only radical scavenger that has been used in clinical practice.^(6)^ Although it had been confirmed that edaravone could exert antioxidative effect via oxidation of itself *in vitro*,^(7)^ the clinical usefulness of other radical scavengers has not been established yet.

**Fig. 1:**
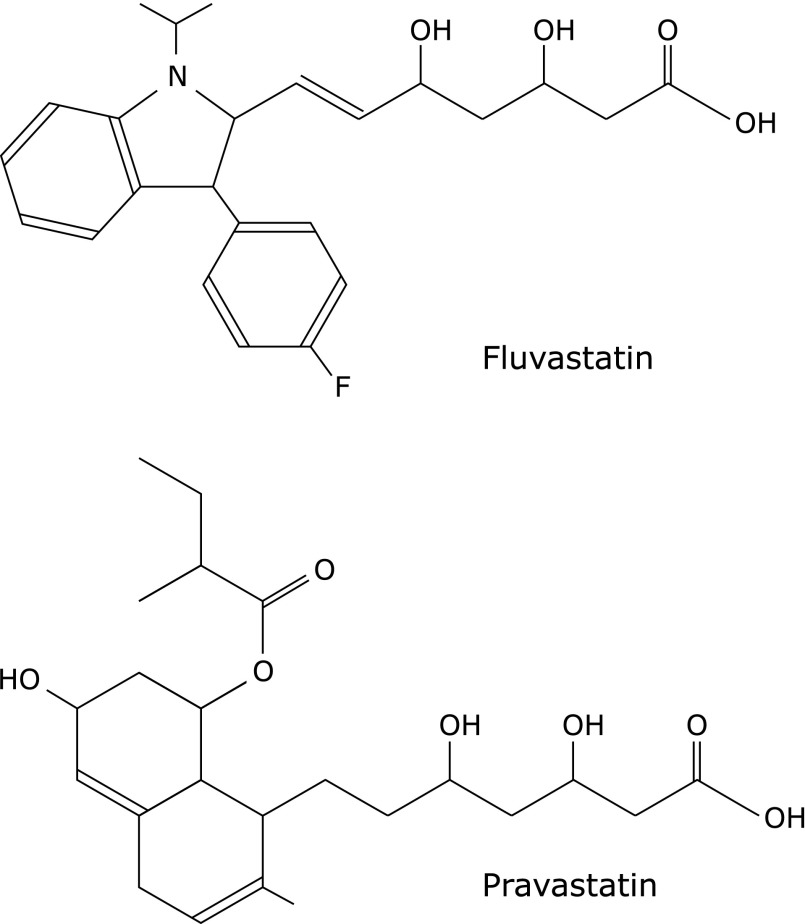
Structure of water-soluble statins, fluvastatin (A) and pravastatin (B).

3-hydroxy-3-methylglutaryl coenzyme A (HMG-CoA) reductase inhibitors, statins, are now widely used for the prevention of cardiovascular or cerebrovascular diseases via lowering serum cholesterol level. It is also known that statins have anti-oxidative effect, which encourages the usage of statins to prevent cerebrovascular and cardiovascular diseases. In fact, several clinical researches revealed such additional benefits of statins to improve patients’ outcome.^(8,9)^ Moreover, several basic researches also showed that statins could reduce hydroxyl radical *in vitro*, or prevent cholesterol-induced oxidative stress in laboratory animals.^(10–12)^ Fluvastatin is a compound with a fluorophenyl indole ring and an enoic acid, and pravastatin is a compound with a naphthalene frame and an enanthate (Fig. 1). Fluvastatin has double bonds in enoic acid conjugated with an indole ring that has shown to have radical scavenging activity,^(13)^ and the same structure is not involved in pravastatin. Therefore, it appears that fluvastatin could have stronger radical scavenging activity than pravastatin. However, the answer to the question as to which of fluvastatin or pravastatin has stronger antioxidative effect has not been clarified yet. In the present study, we compared the scavenging effects of fluvastatin and pravastatin on multiple free radical species including radicals that have not been evaluated yet. For more accurate comparison of the effects of statins on each free radical, we estimated reactive rate constants based on IC_50_ against free radicals obtained.

## Materials and Methods

### Chemicals

Fluvastatin (Wako, Osaka, Japan) and pravastatin (LKT Laboratories Inc., St. Paul, MN) were diluted in distilled water (Milli-Q, Merck Millipore, Darmstadt, Germany) with the concentration ranged from 10 µM to 10 mM, and applied to radical production system described below. 5-(2,2-Dimethyl-1,3-propoxycyclophosphoryl)-5-methyl-1-pyrroline *N*-oxide (CYPMPO) was purchased from Radical Research Inc. (Tokyo, Japan). Hypoxanthine, xanthine oxidase, *tert*-butyl hydroperoxide and 2,2-diphenyl-1-picrylhydrazyl (DPPH) were purchased from Sigma-Aldrich (St. Louis, MO). *N*-methyl-3-(1-methyl-2-hydroxy-2-nitrosohydrazino)-1-propanamine (NOC7) and 2-(4-carboxyphenyl)-4,4,5,5-tetramethylimidazoline-1-oxyl 3-oxide (carboxy-PTIO) were purchased from Dojindo (Kumamoto, Japan). Hydrogen peroxide was purchased from Wako. Other reagents used were of the highest quality commercially available. We used edaravone (Tokyo Chemical Industry, Tokyo, Japan) as a positive control for the scavenging effects on free radicals except for superoxide anion.^(14,15)^ Superoxide dismutase (Sigma-Aldrich, St. Louis, MO) was used as a positive control for the scavenging effects on superoxide anion. All the reagents were freshly prepared at time of use to avoid destabilization, degradation or denaturation of the chemicals due to long-term storage.^(14,15)^

### Free radical measurements

Free radicals were measured using an electron spin resonance (ESR) spectrometer (JES-RE1X ESR, JEOL, Tokyo, Japan) with the spin trapping method.^(16)^ The ultraviolet (UV) light source was a 200 W medium-pressure mercury/xenon arc (RUVF-203SR UV illuminator, Radical Research Inc., Tokyo, Japan) with a UV-transmitting/VIS-absorbing filter (230–430 nm). Free radicals were produced in disposable ESR flat cells (Flash Point Inc., Tokyo, Japan). Instrument settings were as follows: room temperature (23°C); frequency 9.45 GHz with 100-kHz modulation; modulation width 0.1 mT; time constant 0.1 s; sweep time 1 min; center field 335.3 mT. Microwave power was set so that the signals were not saturated (1 to 10 mW). The data were analyzed by an operation software WIN-RAD ver. 1.20b (Radical Research Inc., Tokyo, Japan).

### Free radical production and spin trapping

Each free radical was generated and trapped as previously described.^(16–19)^ Briefly, superoxide anion was produced by the mixture of 0.08 mM hypoxanthine dissolved with phosphate buffer (pH 7.4), 0.04 mM xanthine oxidase, and 2.5 mM CYPMPO as a spin-trap agent.^(17)^ Hydroxyl radical was produced by *in situ* UV irradiation for 4 s to dilute aqueous solution of hydrogen peroxide (0.3%) in a disposable ESR flat cell and trapped by CYPMPO (2.5 mM).^(17)^ *tert*-butyl peroxyl radical, one of alkoxyl radicals, was produced by *in situ* UV irradiation for 10 s to *tert*-butyl hydroperoxide (160 mM) and trapped with 2.5 mM CYPMPO.^(18)^ Nitric oxide radical was produced from NOC7 (250 µM) and reacted with carboxy-PTIO (25 µM) to generate carboxy-PTI, which was measured as an indicator of nitric oxide radical 60 min after the mixture.^(19)^ An artificial radical DPPH (15 µM), which is often used for the assessment of radical scavenging capacity of drugs,^(20)^ was directly observed with ESR.

### Spectrometric analysis and normalization

Intensity of the target signal was measured relative to the external standard of Mn^2+^ and normalized with the control signal (0 mM), and plotted against the concentration of either fluvastatin, pravastatin or edaravone to estimate IC_50_ values of each chemical. In the case of spin trapping method, IC_50_ values are highly dependent upon the free radical production system and the amount of spin trap agent (e.g., CYPMPO, carboxy-PTIO). Therefore, we estimated reaction rate constant (*k*) of each statin (*k*_fv_ for fluvastatin and *k*_pv_ for pravastatin) according to the following equation.^(21)^

$$k_{\text{statin}}=\frac{k_{\text{spin trap }}\text{[spin trap]}}{{\text{IC}}_{\text{50 statin}}}$$

### Statistics

IC_50_ was estimated by sigmoid curve fitting according to Cheng-Prusoff equation. Group averages are presented as mean ± SEM. Differences between group averages were tested using a one-way ANOVA with a post-hoc test (Tukey’s HSD). Significance level was set at 0.05. All statistical analyses were carried out using a statistical software R (ver. 3.4.2).^(22)^

## Results

### Superoxide anion

**Fig. 2:**
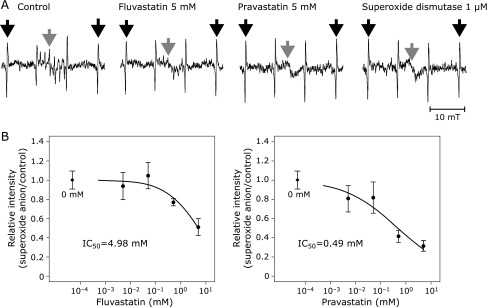
(A) Representative spectra of superoxide anion measured by electron spin resonance spectrometry. Black arrows indicate the external standard of Mn^2+^ and gray arrows indicate the target signals of the radical. (B) The IC_50_ curve of fluvastatin (left) and pravastatin (right) for superoxide anion. Black rhomboids in each graph indicate the relative intensity at 0 mM of each chemical as a reference. Error bars indicate SE of the mean. The number of repetition at each concentration was four.

ESR spectra of superoxide anion were highly diminished by 5 mM fluvastatin and pravastatin (Fig. 2A). Fluvastatin and pravastatin scavenged superoxide anion in a dose-dependent manner with IC_50_ of 4.98 mM and 0.49 mM, respectively (Fig. 2B). Estimated values of *k*_fv_ and *k*_pv_ were 4.82 M^−1^s^−1^ and 49.0 M^−1^s^−1^, respectively (Table 1). Fluvastatin and pravastatin had scavenging activity on superoxide anion, when it was generated by the xanthine-xanthine oxidase system. The value of *k*_pv_ was about ten-times larger than that of *k*_fv_, which suggested that the scavenging activity of pravastatin against superoxide anion was stronger than that of fluvastatin.

### Hydroxyl radical

**Fig. 3:**
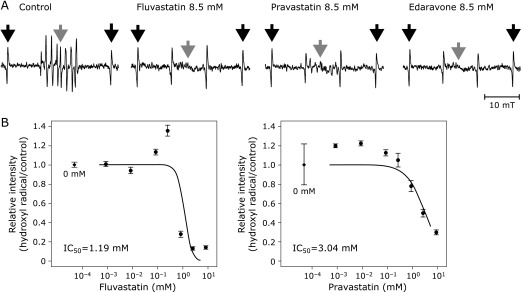
(A) Representative spectra of hydroxyl radical on electron spin resonance spectrometry. Black arrows indicate the external standard of Mn^2+^ and gray arrows indicate the target signals of the radical. (B) The IC_50_ curve of fluvastatin (left) and pravastatin (right) for hydroxyl radical. Black rhomboids in the graphs indicate the relative intensity at 0 mM of each chemical as a reference. Error bars indicate SE of the mean. The number of repetition at each concentration was four.

ESR signals of hydroxyl radical were clearly eliminated by all three compounds (Fig. 3A). Both fluvastatin and pravastatin scavenged hydroxyl radical in a dose-dependent manner with IC_50_ of 1.19 mM and 3.04 mM, respectively (Fig. 3B). Both *k*_fv_ and *k*_pv_ on hydroxyl radical estimated from IC_50_ were >10^9^ M^−1^s^−1^ (Table 1), which indicated that both fluvastatin and pravastatin were highly effective in scavenging hydroxyl radical.

### *tert*-Butyl peroxyl radical

**Fig. 4:**
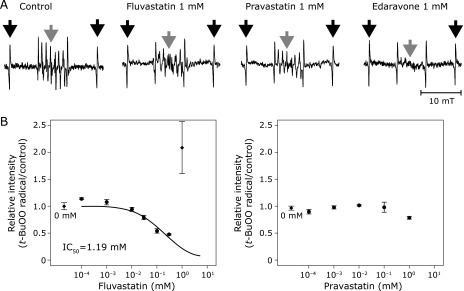
(A) Representative spectra of *tert*-butyl peroxyl radical. Black arrows indicate the external standard of Mn^2+^ and gray arrows indicate the target signals of the radical. (B) The IC_50_ curve of fluvastatin (left) and pravastatin (right) for *tert*-butyl peroxyl radical. Black rhomboids in the graphs indicate the relative intensity at 0 mM of each chemical as a reference. *t*-BuOO: *tert*-butyl peroxyl radical. Error bars indicate SE of the mean. The number of repetition at each concentration was four.

Fluvastatin eliminated *tert*-butyl peroxyl radical, but not in the case of pravastatin (Fig. 4A). Fluvastatin less than 1 mM reduced the signal in a dose-dependent manner with IC_50_ of 0.19 mM (Fig. 4B), which gave us the value of *k*_fv_ (reaction rate constant relative to that of CYPMPO) on *tert*-butyl peroxyl radical as 2.63 (Table 1). However, the signal of *tert*-butyl peroxyl radical in the presence of 1 mM fluvastatin increased up to 2.5 times of that in the absence of fluvastatin (Fig. 4B), suggesting that fluvastatin at high concentration might modulate the generation system of *tert*-butyl peroxyl radical to increase the amount of radical. Pravastatin did not affect *tert*-butyl peroxyl radical at all (Fig. 4A and B).

### Nitric oxide radical

**Fig. 5:**
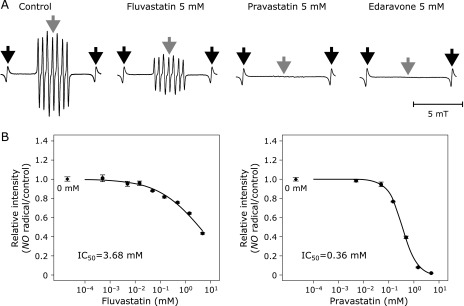
(A) Representative spectra of nitric oxide radical indicated by the amount of carboxyl-PTI. Black arrows indicate the external standard of Mn^2+^ and gray arrows indicate the target signals of the radical. (B) The IC_50_ curve of fluvastatin (left) and pravastatin (right) for nitric oxide radical. Black rhomboids in each graph indicate the relative intensity at 0 mM of each chemical as a reference. NO: nitric oxide. Error bars indicate SE of the mean. The number of repetition at each concentration was four.

Both statins scavenged nitric oxide radical in a dose-dependent manner with IC_50_ of 3.68 mM for fluvastatin and 0.36 mM for pravastatin (Fig. 5A and B). Estimated values of *k*_fv_ and *k*_pv_ on nitric oxide radical were 68.6 M^−1^s^−1^ and 701 M^−1^s^−1^, respectively (Table 1). Pravastatin had ten-times stronger scavenging capacity against nitric oxide radical than fluvastatin.

### DPPH radical

**Fig. 6:**
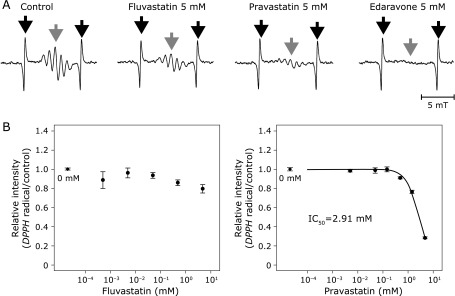
(A) Representative spectra of electron spin resonance spectrometry for DPPH radical. Black arrows indicate the external standard of Mn^2+^ and gray arrows indicates the target signal of the radical. (B) The IC_50_ curve of fluvastatin (left) and pravastatin (right) for DPPH radical. Black rhomboids indicate the relative intensity at 0 mM of each chemical as a reference. Error bars indicate SE of the mean. The number of repetition at each concentration was four.

Representative ESR spectra of DPPH radical are shown in Fig. 5A. Pravastatin scavenged DPPH with IC_50_ of 2.91 mM (Fig. 6B and Table 1). However, fluvastatin did not scavenge DPPH radical.

## Discussion

We evaluated the direct scavenging effects of fluvastatin and pravastatin on multiple free radicals using ESR spectrometry with a configuration of spin trapping method. We found that both fluvastatin and pravastatin had potency to scavenge superoxide anion, hydroxyl radical and nitric oxide radical. On the other hand, *tert*-butyl peroxyl radical was eliminated only by fluvastatin. *tert*-butyl peroxyl radical is an experimental reagent used for simulating alkoxyl radical or peroxyl radical, which is related to oxidation chain reaction of lipids including oxidation of LDL cholesterol as one of the major mechanisms of atherosclerosis. The present result that fluvastatin scavenged *tert*-butyl peroxyl radical suggested that fluvastatin might preventively affect the oxidative chain reaction of lipids, and consequently prevent atherosclerosis via inhibiting oxidation of LDL cholesterol. In fact, Miwa *et al.*^(23)^ had reported that fluvastatin reduced not only oxidative products such as malondialdehyde-modified low-density-lipoprotein (MDA-LDL) but also 3,5,7-cholestatriene in erythrocyte membrane, whereas pravastatin only reduced MDA-LDL. Previously, Nakamura *et al.*^(10)^ clarified that the allylic carbon conjugated with the indole ring of fluvastatin might contribute to the scavenging activity of fluvastatin against *tert*-butyl peroxyl radical. Our present experimental results were consistent with these past reports.

FFA is another source of lipid-derived alkoxyl radical or peroxyl radical *in vivo*. Hara *et al.*^(4)^ showed that FFA could be a marker for oxidative stress as well as antioxidants coenzyme Q-10 in infants with asphyxia. Nagase *et al.*^(5)^ demonstrated that high level of plasma FFA at the onset of ischemia and following decrease in the percentage of unsaturated fatty acid over total FFA significantly correlated with poor outcomes in patients after cardiopulmonary arrest. Basic researches also showed that FFA increased oxidative stress in cultured cells and isolated organs.^(24–26)^ These previous findings indicated that FFA could be a great risk of oxidative stress in several pathophysiological states including ischemia/reperfusion injury. We showed the possibility that fluvastatin may be useful to scavenge lipid-derived free radicals. It was also speculated that fluvastatin could be useful not only for lowering cholesterol but also for preventing oxidative stress caused by FFA during I/R injury in the cases of hypertriglycerolemia for instance.

Nitric oxide radical reacts with superoxide anion and produces nitrogen dioxide or peroxinitrite that has stronger oxidative properties than nitric oxide radical itself. Both nitrogen dioxide and peroxinitrite oxidize nucleic acids, amino acids or lipids to deteriorate their functions.^(27,28)^ On the other hand, nitric oxide radical exerts vasodilator action via cyclic guanidine monophosphate (cGMP) activation.^(29,30)^ We confirmed that nitric oxide radical was significantly reduced by both fluvastatin and pravastatin, and the effect of pravastatin was more potent than that of fluvastatin. Moreover, pravastatin had stronger scavenging effects on superoxide anion than fluvastatin, which was suggested from higher value of *k*_pv_ than that of *k*_fv_. From these results, pravastatin could prevent the production of nitrogen dioxide and peroxinitrite more potently than fluvastatin via stronger scavenging activity against both superoxide anion and nitric oxide radical. However, we also have to consider that the reduction of nitric oxide radical by statins may impair vasodilation. Further experiments using cultured cells or laboratory animals are still required to conclude that the deletion of nitric oxide by statins *in vivo* would be beneficial or harmful.

We demonstrated that both fluvastatin and pravastatin had scavenging effects on multiple radicals, whereas *tert*-butyl peroxyl radical was eliminated only by fluvastatin and DPPH radical was scavenged only by pravastatin. We also evaluated the effects of fluvastatin and pravastatin on superoxide anion using the experimental system of ultraviolet irradiation to concentrated hydrogen peroxide^(17)^ and found that radical was significantly reduced only by fluvastatin but not by pravastatin (Supplemental Fig. 1*). We considered that the discrepancy was caused by the photochemical properties of the phenol group of fluvastatin.^(31)^ Furthermore, we first evaluated the scavenging effects of statins on superoxide anion in xanthine-xanthine oxidase system using 5,5-Dimethyl-1-pyrroline 1-oxide (DMPO, purchased from Dojindo, Kumamoto, Japan) as a spin trapping agent and found that both statins had less effects on the radical (Supplemental Fig. 2*). However, when CYPMPO was used as a spin trapping agent for the same experimental setting, we could clearly reveal the scavenging effects of both statins on superoxide anion (Fig. 1). DMPO is often used as a spin trapping agent to detect oxygen-centered radicals. However, the previous study revealed that CYPMPO had a larger reaction rate constant with superoxide anion than DMPO and the superoxide adduct of CYPMPO was more stable than that of DMPO.^(17)^ Thus, the authors concluded that it would be better to use CYPMPO rather than DMPO for the detection of superoxide anion in the present study. When evaluating the antioxidant effects of chemicals, it is possible that a conclusion with only a single experimental system may lead to a misinterpretation. It would be important to evaluate the antioxidative reaction of the chemicals with multiple radicals and multiple experimental systems.

## Limitations

The present study only evaluated two kinds of water-soluble statins, fluvastatin and pravastatin. In clinical practice, other lipophilic statins (e.g., atorvastatin, pitavastatin, simvastatin, etc.) are commonly used, and those drugs are also known as potent anti-oxidants,^(32,33)^ which were not assessed in the present study. To apply ESR spectroscopy to those drugs, it is necessary to dilute them in an organic solvent such as dimethyl sulfoxide or alcohol. However, such solvents could scavenge free radical species or react with them by themselves, thus it could be difficult to assess the effect of lipophilic statins on free radicals using ESR spectroscopy.

Another factor might be possible equilibrium in the statin solutions. In case of edaravone, it is present as unstable edaravone anion in aqueous solution, which is capable of transferring an electron to (i.e., reducing) free radicals and becomes edaravone radical.^(14,15)^ We prepared all the reagents freshly at time of use to prevent their denaturation and degradation, and mechanisms involving such equilibrium is not considered in the present discussion.

Fluvastatin and pravastatin reduced multiple free radical species *in vitro*. Both fluvastatin and pravastatin had scavenging effects on superoxide anion, hydroxyl radical and nitric oxide. On the other hand, *tert*-butyl peroxyl radical was scavenged only by fluvastatin, suggesting that fluvastatin might have more potential effect than pravastatin to prevent atherosclerosis and ischemia/reperfusion injury via inhibiting oxidation of lipids.

## Author Contributions

Umeda R, Acquisition, analysis and interpretation of the data. Statistical analysis. Drafting of the manuscript. Takanari H, Study concept and design. Acquisition, analysis and interpretation of the data. Statistical analysis. Drafting of the manuscript. Obtained funding. Material support. Ogata K, Acquisition, analysis and interpretation of data. Technical support. Matsumoto S, Obtained funding. Material support. Kitano T, Obtained funding. Material support. Ono K, Study supervision, Tokumaru O, Study concept and design. Acquisition, analysis and interpretation of the data. Drafting of the manuscript. Critical revision of the manuscript. Obtained funding. Material and technical support.

## Figures and Tables

**Table 1:** Relative reaction rate constants for fluvastatin (*k*_fv_) and pravastatin (*k*_pv_)

Free radical	hfsc^†^ (mT)	Statin	IC_50_ (mM)	Reaction rate constant (M^−1^s^−1^)
	a_H_, a_N_, a_P_			
Superoxide anion	1.04, 1.32, 5.11	fluvastatin	4.98	4.82
	1.15, 1.30, 5.24	pravastatin	0.49	49
Hydroxyl radical	1.40, 1.40, 5.04	fluvastatin	1.19	>10^9^
	1.22, 1.40, 4.90	pravastatin	3.04	>10^9^

*tert*-Butyl peroxyl radical	1.35, 1.45, 4.95	fluvastatin	0.19	2.63 × *k*_CYPMPO_^‡^
		pravastatin	no effect	—

Nitric oxide	a_N1_ 0.981,	fluvastatin	3.68	68.6
	a_N2_ 0.445	pravastatin	0.36	701

DPPH^§^	—, 0.903, —	fluvastatin	no effect	—
		pravastatin	2.91	—

^†^Hyperfine splitting constant. ^‡^Value unknown. ^§^2,2-diphenyl-1-picrylhydrazyl.

## Abbreviations

carboxy-PTIO: 2-(4-carboxyphenyl)-4,4,5,5-tetramethylimidazoline-1-oxyl 3-oxide
cGMP: cyclic guanidine monophosphate
CYPMPO: 5-(2,2-Dimethyl-1,3-propoxycyclophosphoryl)-5-methyl-1-pyrroline *N*-oxide
DMPO: 5,5-Dimethyl-1-pyrroline 1-oxide
DPPH: 2,2-diphenyl-1-picrylhydrazyl
ESR: electron spin resonance
FFA: free fatty acid
HMG-CoA: 3-hydroxy-3-methylglutaryl coenzyme A
IC_50_: 50% inhibitory concentration
I/R: ischemia/reperfusion
MDA-LDL: malondialdehyde-modified low-density-lipoprotein
NOC7: *N*-methyl-3-(1-methyl-2-hydroxy-2-nitrosohydrazino)-1-propanamine
UV: ultraviolet
