# Supplementary material for: Direct free radical scavenging effects of water-soluble HMG-CoA reductase inhibitors
Source: J Clin Biochem Nutr. 2018 Aug 8;64(1):20–6. doi: 10.3164/jcbn.18-48 (PMC6348410; doi:10.3164/jcbn.18-48)
Supplement: Supplemental Table 1 [file jcbn18-48sf01.pdf]

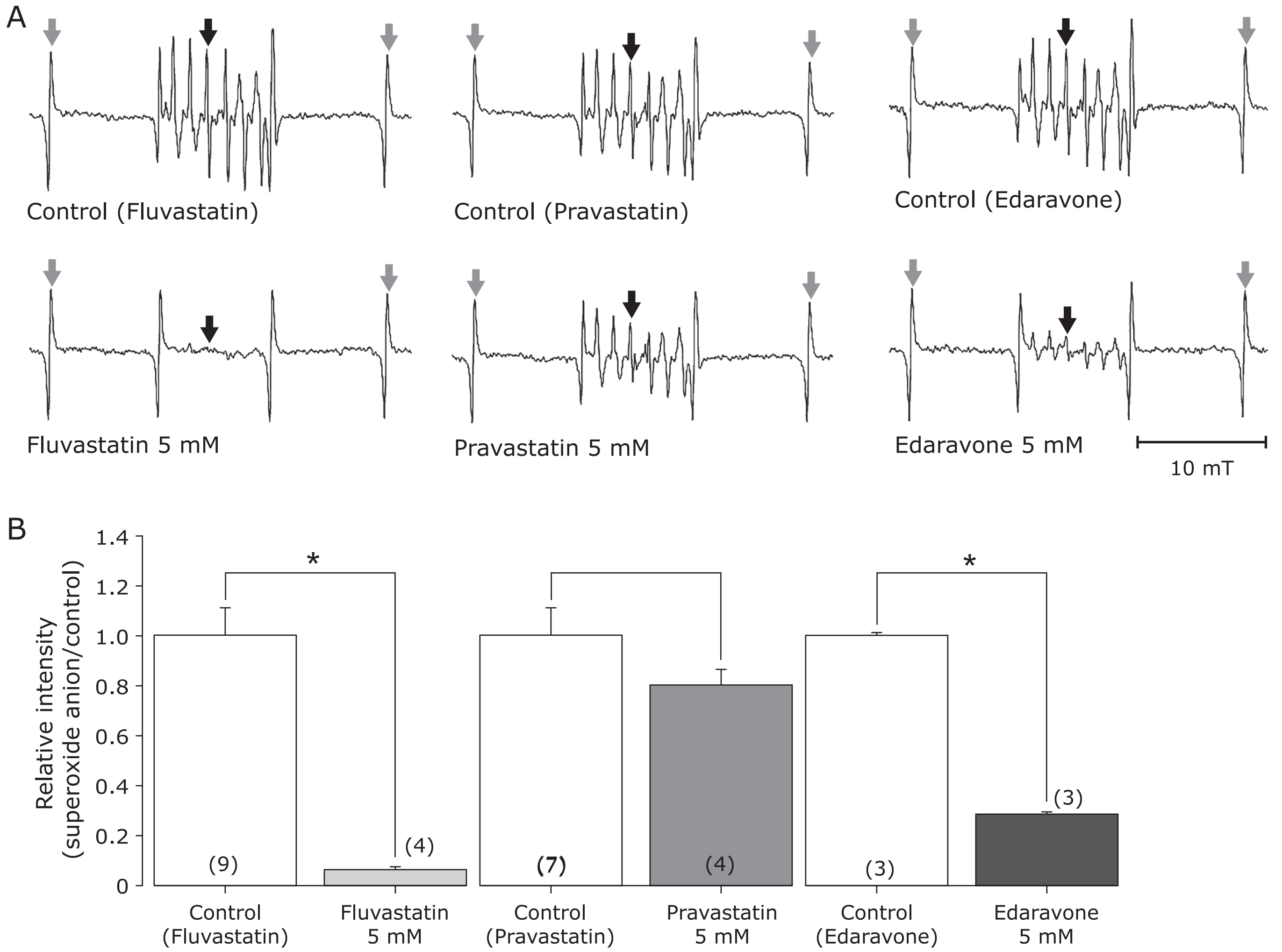

Supplemental Fig. 1. (A) Representative ESR spectra of superoxide anion generated by ultraviolet irradiation to concentrated hydrogen peroxide. Gray arrows indicate the external standard signals of Mn^{2+} and black arrows indicate the measured target signals of the radical. (B) Summarized data of the intensity of superoxide. Blank control; light gray 5 mM fluvastatin; dark gray 5 mM pravastatin; black 5 mM edaravone (positive control). The number in parenthesis indicates the number of repetition at each concentration. *p<0.001.
