# Supplementary material for: Direct free radical scavenging effects of water-soluble HMG-CoA reductase inhibitors
Source: J Clin Biochem Nutr. 2018 Aug 8;64(1):20–6. doi: 10.3164/jcbn.18-48 (PMC6348410; doi:10.3164/jcbn.18-48)
Supplement: Supplemental Table 2 [file jcbn18-48sf02.pdf]

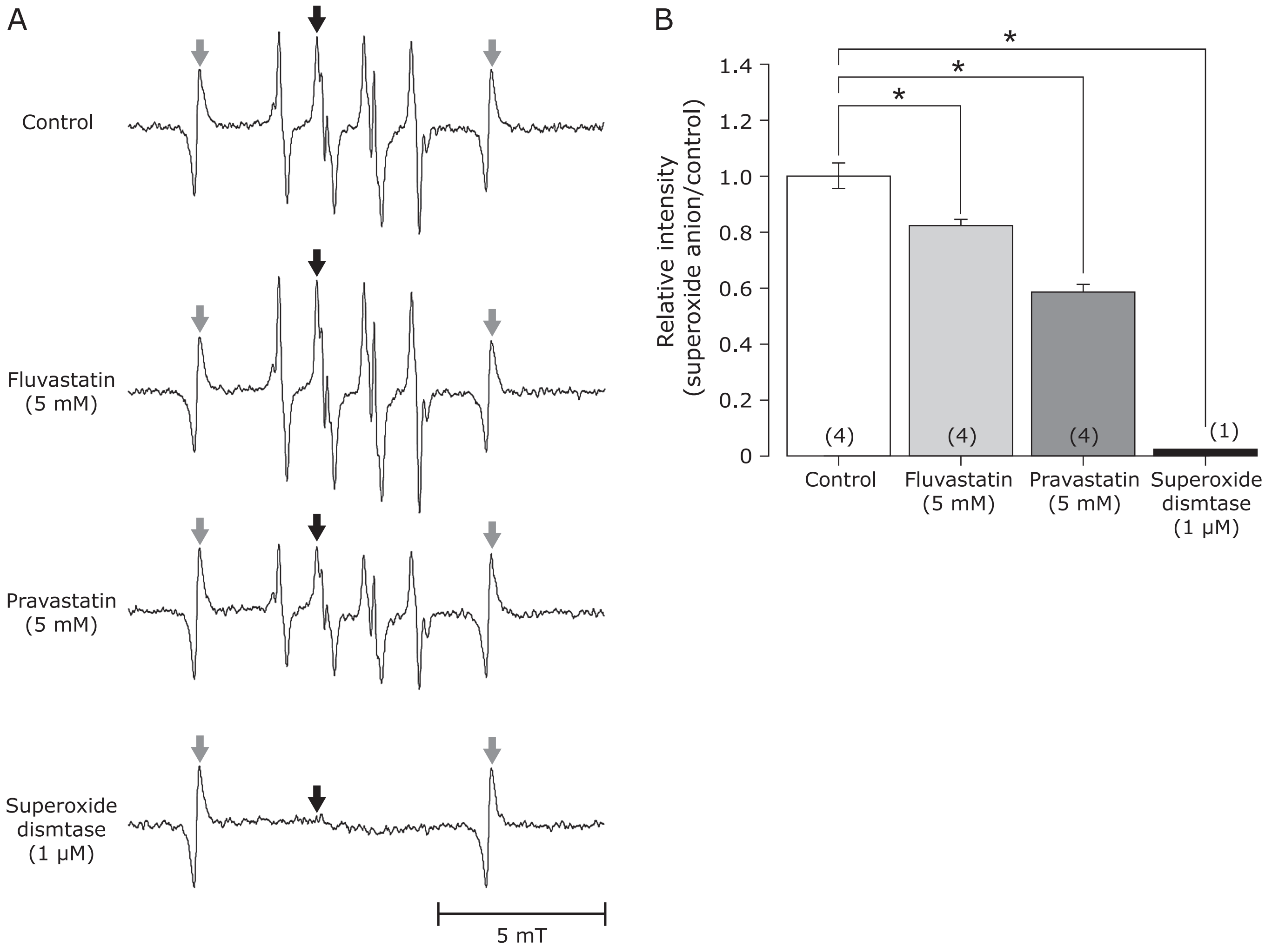

Supplemental Fig. 2. (A) Representative spectra of superoxide anion generated by xanthine-xanthine oxidase system with DMPO. Gray arrows indicate the external standard signals of Mn^{2+} and black arrows indicate the measured target signals of the radical. (B) Summarized data of intensity of superoxide anion. Blank control; light gray 5 mM fluvastatin; dark gray 5 mM pravastatin; black 1 μM superoxide dismutase (positive control). The number in parenthesis indicates the number of repetition at each concentration. *$p<0.001$.
